# Excess all-cause mortality and COVID-19 reported fatality in Iran (April 2013–September 2021): age and sex disaggregated time series analysis

**DOI:** 10.1186/s13104-022-06018-y

**Published:** 2022-04-05

**Authors:** Seyed Amir Ahmad Safavi-Naini, Yeganeh Farsi, Walid Q. Alali, Ali Solhpour, Mohamad Amin Pourhoseingholi

**Affiliations:** 1 National Research Institute of Tuberculosis and Lung Diseases, Shahid Beheshti University of Medical Sciences, Tehran, Iran; 2 Student’s Research Committee, School of Medicine, Shahid Beheshti University of Medical Sciences, Tehran, Iran; 3 Department of Epidemiology & Biostatistics, Faculty of Public Health, Kuwait University, Kuwait, Kuwait; 4 Department of Anesthesiology, University of Florida, Gainesville, USA; 5 Basic and Molecular Epidemiology of Gastrointestinal Disorders Research Center, Research Institute for Gastroenterology and Liver Diseases, Shahid Beheshti University of Medical Sciences, Tehran, Iran

**Keywords:** COVID-19, Mortality, Cause of death, Iran, Interrupted time series analysis

## Abstract

**Objective:**

The actual impact of the pandemic on COVID-19 specific mortality is still unclear due to the variability in access to diagnostic tools. This study aimed to estimate the excess all-cause mortality in Iran until September 2021 based on the national death statistics.

**Results:**

The autoregressive integrated moving average was used to predict seasonal all-cause death in Iran (R-squared = 0.45). We observed a 38.8% (95% confidence interval (CI) 29.7%–40.1%) rise in the all-cause mortality from 22 June 2020 to 21 June 2021. The excess all-cause mortality per 100,000 population were 178.86 (95% CI 137.2–220.5, M:F ratio = 1.3) with 49.1% of these excess deaths due to COVID-19. Comparison of spring 2019 and spring 2021 revealed that the highest percent increase in mortality was among men aged 65–69 years old (77%) and women aged 60–64 years old (86.8%). Moreover, the excess mortality among 31 provinces of Iran ranged from 109.7 (Hormozgan) to 273.2 (East-Azerbaijan) per 100,000 population.

In conclusion, there was a significant rise in all-cause mortality during the pandemic. Since COVID-19 fatality explains about half of this rise, the increase in other causes of death and underestimation in reported data should be concerned by further studies.

**Supplementary Information:**

The online version contains supplementary material available at 10.1186/s13104-022-06018-y.

## Introduction

In recent years, cardiovascular diseases, cancers, and injuries have been the leading causes of death worldwide [1, 2]. In Iran, cardiovascular disease (44%), Cancers (17%), and kidney disease (7%) were the most common cause of death, followed by road injury (6%), which declined in recent years [3].

The ongoing COVID-19 pandemic drastically challenged our healthcare system, caused over 4.5 million deaths worldwide, and has considerably impacted all-cause excess mortality [2, 4, 5]. The lack of adequate health system infrastructure, inequity in healthcare accessibility, poor public health education, decentralized decision making, and the nature of SARS-CoV-2 as an emerging virus resulted in increased mortality during the COVID-19 pandemic [6–8]. Excess all-cause mortality would be a better indicator of COVID-19 impact on the health care system than the COVID-19 reported mortality rate. It provides a comparing context between different management strategies and can guide policy-making strategies more efficiently [9, 10]. There was an increase in the all-cause mortality rate (excess death) in Iran since winter 2020 compared to the expected mortality rate based on previous year data, likely attributed to the COVID-19 pandemic [11, 12]. Nonetheless, the real impact of the COVID-19 pandemic on mortality is still unclear, as it can be easily affected by the availability and accessibility of diagnostic tools and reporting systems [10, 13]. This study aimed to investigate the gap between predicted mortality and observed mortality from the start of the pandemic in Iran from January 2020 to June 2021 based on the national statistics of mortality.

## Material and methods

### Data set

This study used publicly available data provided by the Ministry of Health and Medical Education (MOHME) and Iran's National Organization of Civil Registration (NOCR). A daily number of COVID-19 cases and mortality reported by MOHME was gathered from Iran Data Administration (IRDA) [14]. Weekly all-cause death counts from 21 March 2013 to 22 September 2021 were collected from the NOCR website for 31 provinces of Iran (variables: Gender, Death Count, Date, Province, 5-year increment age group) [15]. Additional File 1: Figure S1 shows the data flow in Iran’s national death registration system. The national death registration program of NOCR and MOHME gather the mortality data from provincial health centers. Jafari et al. described this program in more detail [16]. We calculated the 2020 population of Iran for the 31 provinces based on the 2011 and 2016 census and assumed that the mode of increase observed between two dates in the past has been constant. The 2021 Iranian population was used to estimate the age-specified all-cause mortality rate and all-cause mortality rate per 100,000 population.

### Statistical analysis

Expected seasonal mortality and 95% confidence interval (CI) from winter 2019 until spring 2021 was estimated based on a time series analysis of seasonal mortality of spring 2013 to autumn 2019. The time series modeler selected the best-fitting Auto-Regressive Integrated Moving Average (ARIMA) or exponential smoothing model for forecasting-based Bayesian Criterion Information (BIC) measure. Seasonal additive effect and outliers were considered for time series analysis. The forecasting was carried out for all-cause seasonal deaths among men and women, by age group, and for the total population for the 31 provinces of Iran.

Excess death (with 95% CI) was considered as the gap of expected (with 95% CI) and observed seasonal deaths. The excess death rate per 100,000 population was measured by the proportion of excess deaths divided by the specified population. The total excess death of the year from summer 2020 to spring 2021 was then evaluated. We excluded the winter 2020 excess death data because of the possible immaturity of the COVID-19 reporting system. The mortality gap between spring 2019 and spring 2021 was estimated to define the age-specified excess mortality rate. Then by using the 5-year age group population of 2021 in Iran, the age-specified excess mortality rate was calculated [17]. The percent change of each 5-year age group was provided comparing spring 2019 and spring 2021.

R software version 3.6.3 was used for the statistical analysis. Linear regression was performed to evaluate the relation of weekly all-cause death with weekly COVID-19 death. Durbin-Watson statistic to check the independence of observations. The significance level was considered as P < 0.05.

### Results

The ARIMA model was the best-fitted model for male, female, and total population at the national level. Additional file 1: Table S1 shows the model fit statistics for forecast models among genders and provinces. The selected model for mortality at national level was ARIMA (p = 0, d = 0, q = 0) (sp = 0 ,sd = 1, sq = 0) which is especial case of ARIMA named random walk model.

**Fig. 1:**
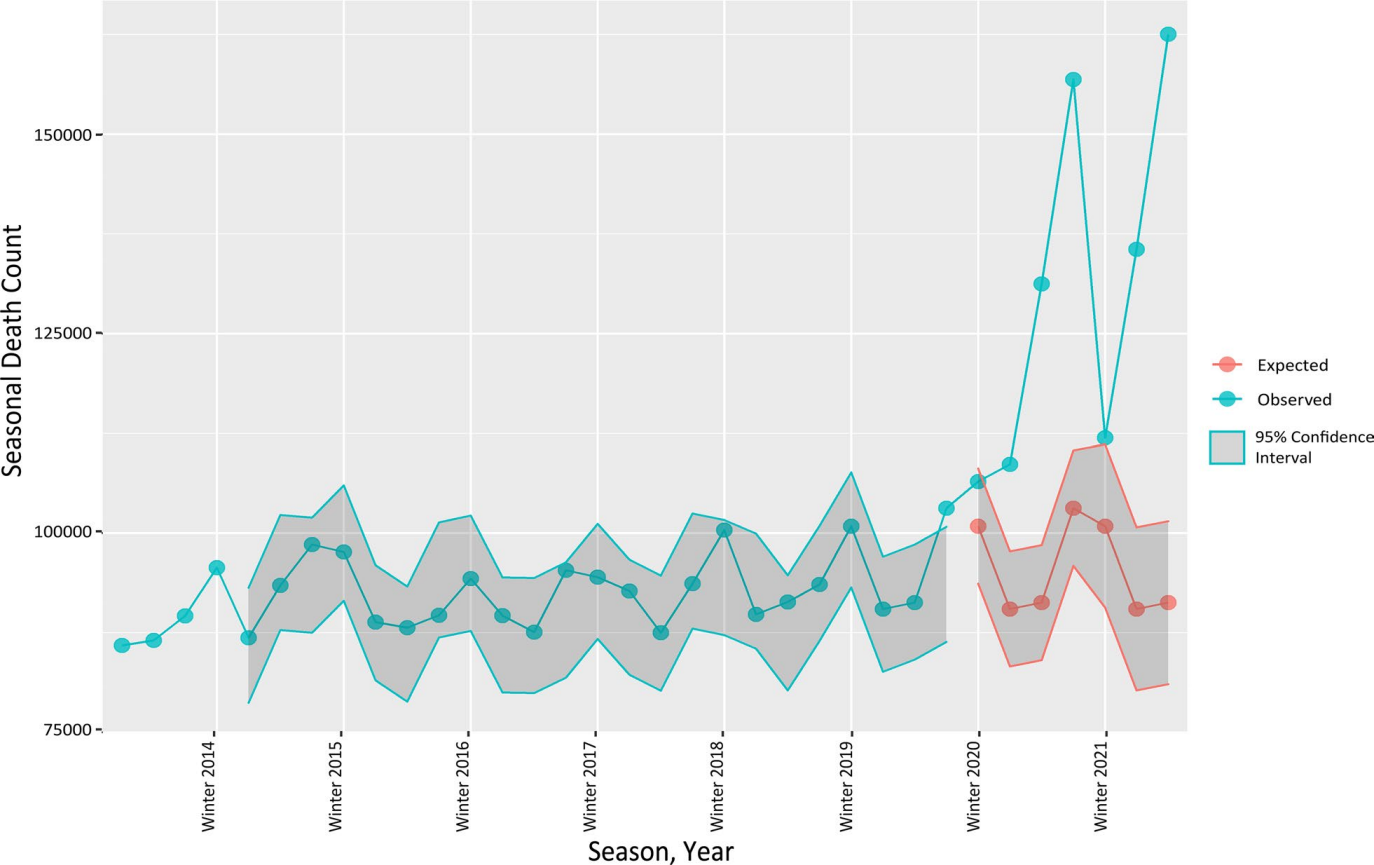
Observed versus expected seasonal mortality from spring 2013 to summer 2021 in Iran

As Fig. 1 depicted, based on the prediction of all-cause mortality, the expected death count was 385,778 deaths for summer 2020 until spring 2021. However, 535,570 deaths occurred in Iran during the study period, 38.8% (95% Confidence Interval (CI) 29.7%–40.1%) higher than the predicted value. As shown in Additional file 2: Table S2, the rise in deaths is evident in all seasons during the pandemic and was higher among the male population. From summer 2020 to spring 2021, the excess deaths per 100,000 population was 178.86 (95% CI 137.20–220.51) with a male to female ratio of 1.3. In addition, summer 2021 showed the highest excess death of 85.13 (95% CI 97.33–72.93) per 100,000 population. COVID-19 reported fatality accounted for 49.1% of excess deaths, and it ranges from 22.9% in winter 2019 to 73.4% in winter 2020 during the pandemic (Additional file 2: Table S2).

**Fig. 2:**
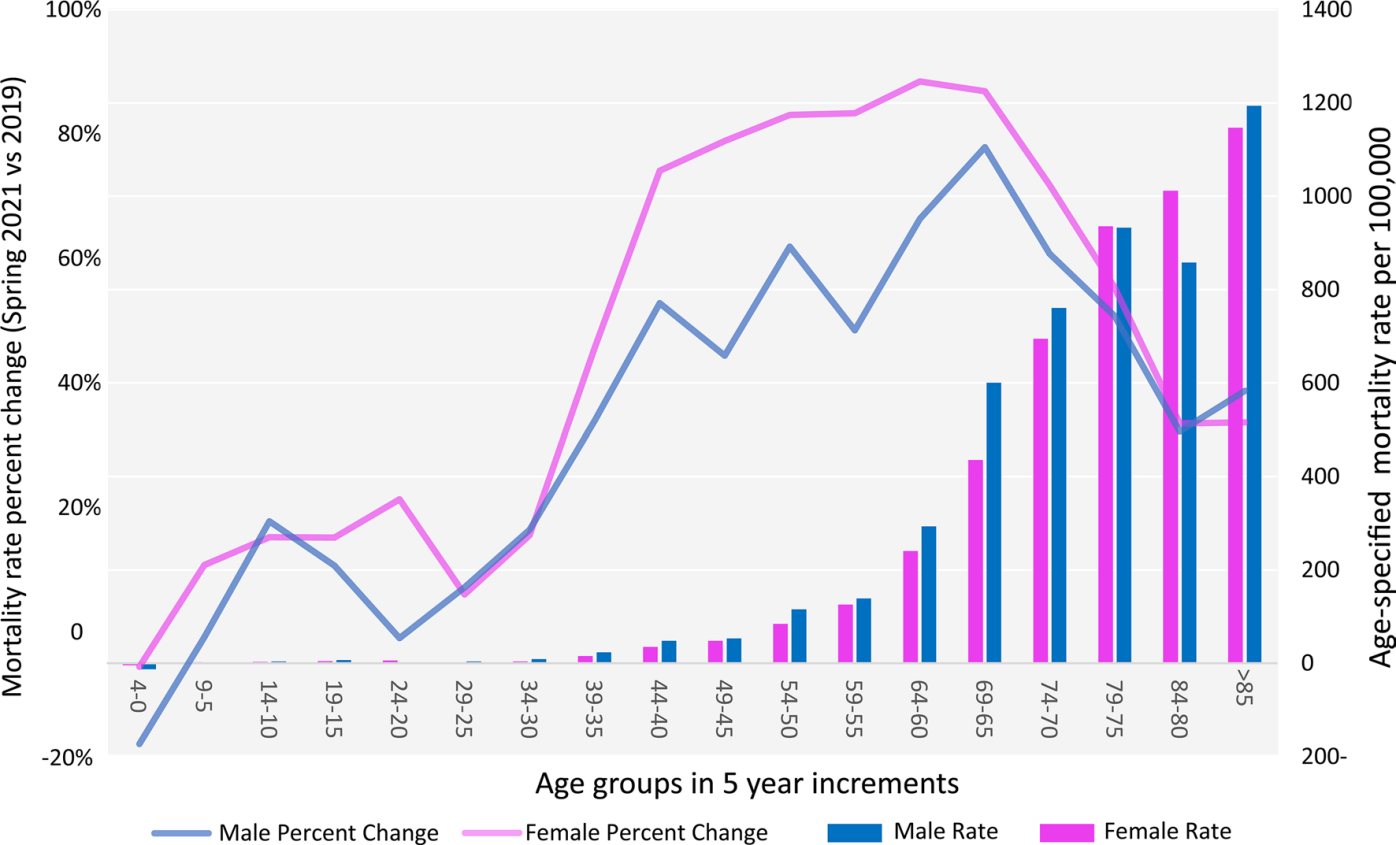
Age specified mortality rate (gap of spring 2021 compared to spring 2019) among males and females

Figure 2 shows the 2019 spring and 2021 spring mortality gap among 5-year age groups. The age-specific mortality per 100,000 population older than 85 years was the highest mortality, compared to other age groups. Moreover, the highest percent change was among men, aged from 65 to 69 years old (77%), and women, aged from 60 to 64 years old (86.8%). The mortality percent rise in the 10–20 years old age group (14.7%) was higher than the 20–30 years old population (8.4%). Bimodal age pattern was presented with a low peak in the 10–20-year age group followed by a significant rise in the 60–70 years old group.

The weekly all-cause deaths, COVID-19 reported, and COVID-19 said cases from 23 March 2019 until 22 September 2021 are shown in Additional file 3: Figure S2. Weekly COVID-19 reported deaths coverage of all-cause death ranged from 4 to 32%. There was a statistically significant relationship between COVID-19 death and all-cause death as demonstrated in Additional file 3: Figure S3 (P-value = 0.001, Beta coefficient 95% CI  1.57–2.06, R-squared = 0.75).

**Fig. 3:**
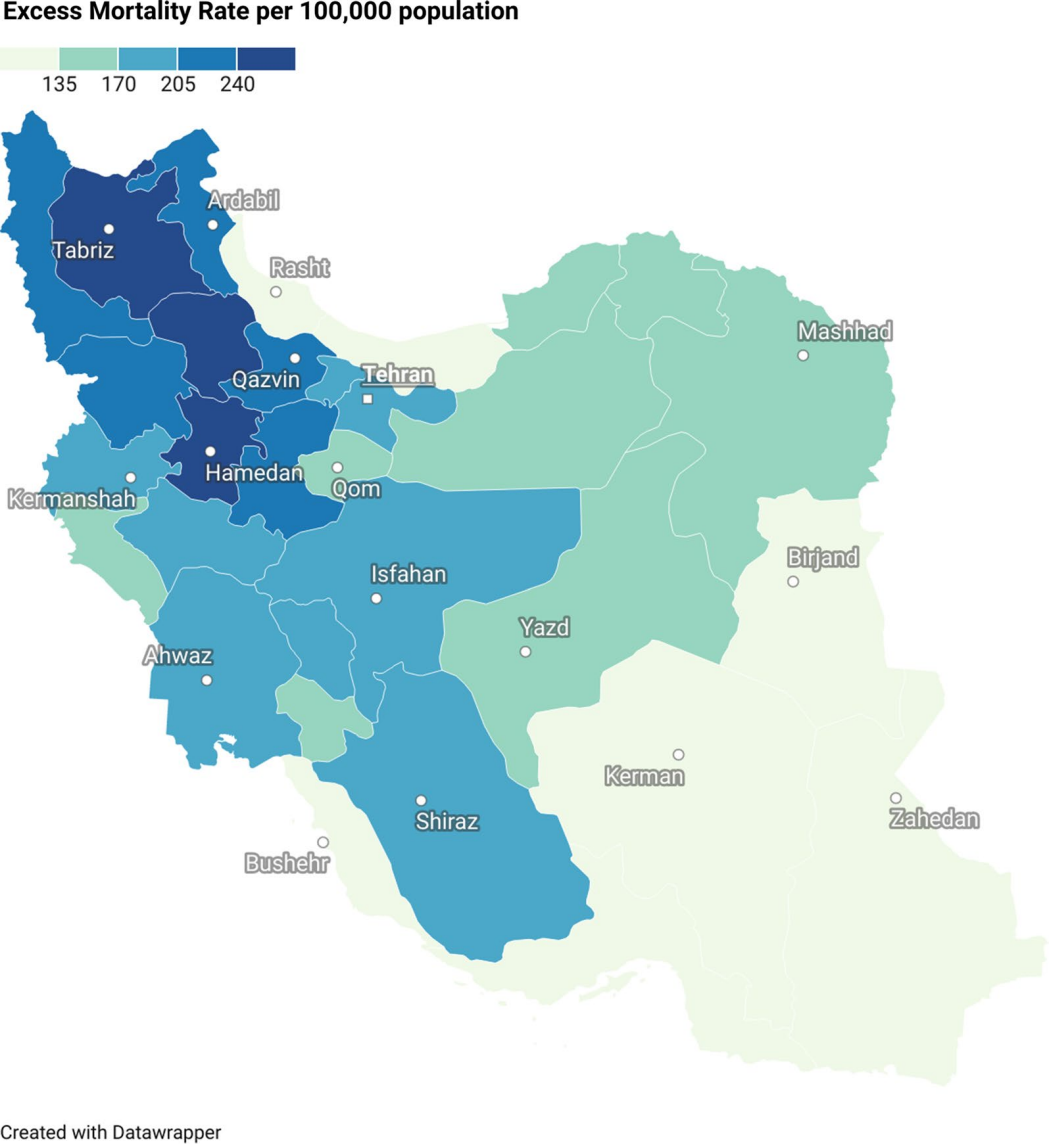
Density map of all-cause mortality rate per 100,000 population among 31 provinces of Iran in a year from summer 2020 to spring 2021

As presented in Fig. 3, all 31 provinces of Iran showed a rise in all-cause mortality. Men’s excess death rate was not statistically significantly different among regions (P-value = 0.09, mean difference 95% CI: −3.9 to 49.7). Excess death rate ranged from 109.7 (Hormozgan) to 273.2 (East Azerbaijan) per 100,000 population and showed higher rates among men than women in 26/31 provinces (Additional file 2: Table S3).

## Discussion

Investigations of mortality statistics worldwide have demonstrated increased mortality rates during the COVID-19 pandemic compared to previous years [18, 19]. While the COVID-19 pandemic has an undeniable impact on excess mortality, it should be highlighted that the rise in mortality linked to COVID-19 is not the only explanation for the increased total mortality [20–22]. Our age and sex-disaggregated time series analysis of seasonal mortality from summer 2020 to spring 2021 in Iran revealed an excess all-cause mortality rate of 178.8 (95% CI 137.2–220.5) per 100,000 population with a higher increase among males and older adults. Inconsistent with other studies, COVID-19 fatality was significantly related to all-cause excess mortality [23]. The excess death rate in the USA, the UK, Italy, and Spain was 136, 121, 179, and 166 (per 100,000 population), aligning with the excess death rate of 178 in Iran [23].

The percent of excess deaths attributed to COVID-19 (calculated by dividing COVID-19 deaths by excess deaths) varies worldwide. While the COVID-19 ends explain more than half of excess deaths in the USA (77%), UK (87%), Italy (67%), Spain (65%), and Brazil (79%); the percent of excess deaths attributed to COVID-19 was low in Kazakhstan (9%), Russia (16%), Kyrgyzstan (20%), Ecuador (36%) [23]. The malignancies and cardiac-associated mortalities, the leading causes of mortality in recent years, have also increased during the pandemic, mainly due to public fear of referring to hospitals and delay in critical state management [24–29]. Since we found half of the excess deaths in Iran have been attributed to COVID-19, the underreporting and rise in other causes of death may explain the other half. Causes of deaths in Iran during the pandemic are not available; further studies should assess the trend of other causes of death during the pandemic.

The gap of excess mortality is more prominent in males in almost all countries. Inconsistent with other studies, we found a male to female excess death ratio of 1.3, and nearly all provinces had higher excess mortality among the male population than females (Additional file 2: Table S3). COVID-19 affects the male population and older adults the most. Therefore, the significant change during the pandemic among male and older people can be explained by the impact of COVID-19 [30].

In addition, our results showed 60–70 years-old population was influenced the most and had the highest excess death percent increase (Fig. 2). Other studies also showed a similar age pattern [31]. It is also noteworthy to imply that we have found the 65–69 years old males and 60–64 years old females to be the most affected age groups by COVID-19. There is a discrepancy among the studies from different countries about the most affected age group. Although the number of infections was the highest among the 20–40-year age group in India, South Korea, and the USA [32, 33], the highest number of infections in China and Italy were in elderlies [34]. We observed 10–20 years-old age group had a slightly higher excess death than the 20–30 years-old group. Further studies are helpful to evaluate the cause of this slight peak within the 10–20 years-old age group.

All provinces of Iran showed a rise in the total number of deaths during the pandemic, but the excess death rate varies widely among areas; excess end ranges from 273.2 in East Azerbaijan to 99.2 in Hormozgan per 100,000 population. The death census in Iran faces underreporting and late reporting of death data [35]. The disruption of registrations during the pandemic may cause higher underreporting, especially in under-developed provinces. Since many areas with majority Turk ethnicity (East Azarbaijan, Zanjan, Ardabil, West, Azarbaijan) showed high excess mortality, the race may explain some extent of this variation [36]. The current literature has not evaluated the risk of COVID-19 mortality among ethnic groups in the Iranian population to our best knowledge.

In conclusion, there was a significant rise in all-cause mortality during the pandemic, especially in older adults and the male gender. Although COVID-19 fatality explains some extent of the increase in all-cause deaths, the increase in other causes of death and underestimation in reported data should be concerned by further studies.

## Limitations

There are many limitations to this study. While using time-series analysis to predict future data points can provide insights, they cannot give a precise forecast of the future. Furthermore, the different causes of deaths during or before the pandemic were unavailable in Iran. Moreover, COVID-19 fatality was not available by age or sex in each province or just for the total population. These datasets can provide a better evaluation of the COVID-19 pandemic impact. The death registration may get disrupted by the pandemic itself as there were some previous issues in underreporting and late registration of deaths, especially in low-resource provinces.

## Supplementary Information

**Additional file 1: Figure S1.** The flow of mortality data through Iran’s death registration system. **Table S1.** The prediction model and fitness statistics are based on seasonal deaths from 2013 to autumn 2019 in Iran and Iran’s provinces.

**Additional file 2: Table S2.** The seasonal excess death rate, male to female ratio, and COVID-19 reported deaths coverage from winter 2020 to summer 2021. **Table S3.** Excess deaths at the province level in a year from summer 2020 until spring 2021 in Iran.

**Additional file 3: Figure S2.** Weekly all-cause death, COVID-19 reported death, and COVID-19 reported cases from 23 March 2019 to 22 September 2021. **Figure S3.** Relation of weekly all-cause death and COVID-19 reported mortality during the COVID-19 pandemic in Iran (From March 2020 until September 2021)

## Data Availability

The datasets supporting the conclusions of this article are (are) available in Iran’s National Civil Registration office repository, https://www.sabteahval.ir/Page.aspx?mId=49826&ID=3273&Page=Magazines/SquareshowMagazine.

## Abbreviations

COVID-19: Coronavirus disease 2019
MOHME: Ministry of health and medical education
NOCR: National organization of civil registration
CI: Confidence interval
ARIMA: Autoregressive integrated moving average
BIC: Bayesian criterion information
