# Supplementary material for: Excess all-cause mortality and COVID-19 reported fatality in Iran (April 2013–September 2021): age and sex disaggregated time series analysis
Source: BMC Res Notes. 2022 Apr 5;15:130. doi: 10.1186/s13104-022-06018-y (PMC8981187; doi:10.1186/s13104-022-06018-y)
Supplement: Supplementary file 1 — Additional file 1: Figure S1. The flow of mortality data through Iran’s death registration system. Table S1. The prediction model and fitness statistics are based on seasonal deaths from 2013 to autumn 2019 in Iran and Iran’s provinces. [file 13104_2022_6018_MOESM1_ESM.pdf]

Figure S1. Flow of mortality data through Iran's death registration system

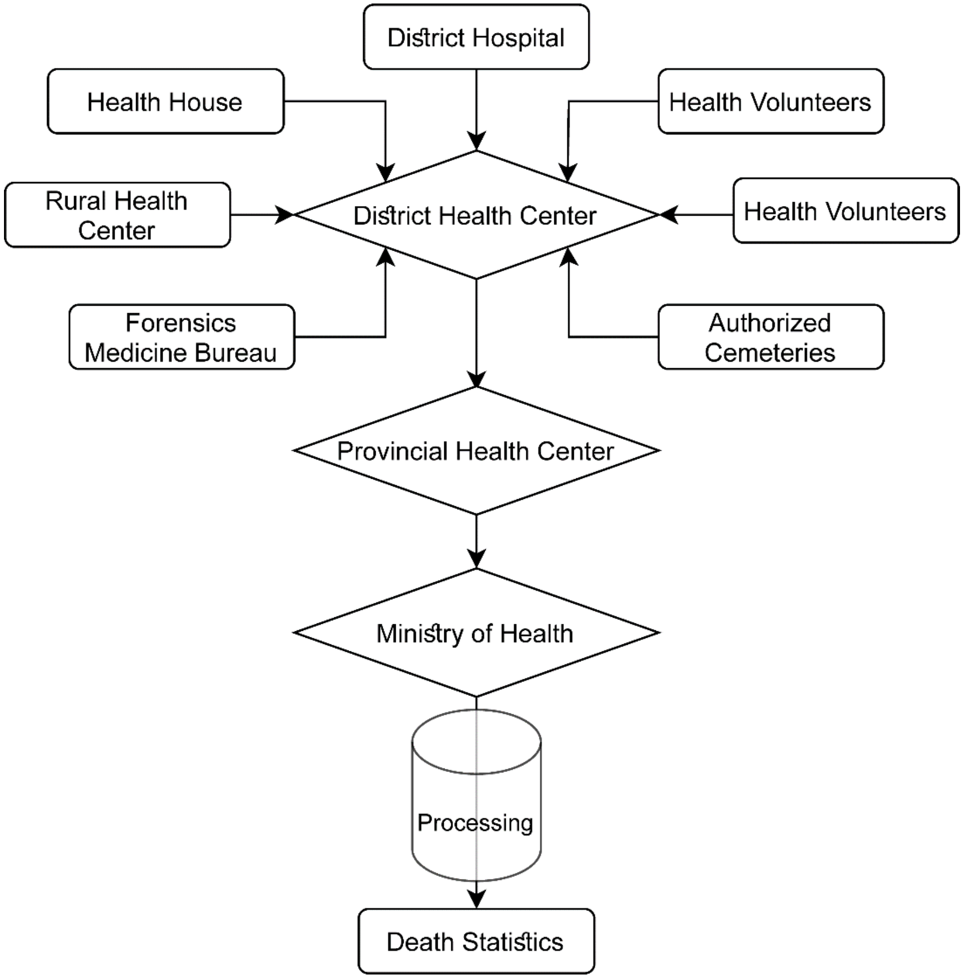

Table S1. Type of prediction model and fitness statistics based on seasonal deaths from 2013 to autumn 2019 in Iran and Iran's provinces.

		Model Fit statistics			Ljung-Box Q(18)		Sig.	No. Outliers
Name of province	Model type (p,d,q) (sp,sd,sq)	Stationary R-squared (SD)	R-squared (SD)	Normalized BIC (SD)	Statistics	DF		
All models		0.62 (0.24)	0.58 (0.25)	9.08 (1.75)				
**National (Iran)**	ARIMA(0,0,0)(0,1,0)	0.39	0.45	16.58	32.78	18.00	0.02	1
Azarbaijan_Sharghi	Simple Seasonal	0.78	0.34	11.17	13.23	16.00	0.66	0
Azarbaijan_Gharbi	ARIMA(0,0,0)(0,1,0)	0.92	0.83	10.51	20.49	18.00	0.31	2
Ardebil	ARIMA(1,0,0)(0,1,0)	0.49	0.07	9.74	19.37	17.00	0.31	1
Esfahan	Winters' Additive	0.64	0.55	11.25	28.50	15.00	0.02	0
Alborz	ARIMA(0,0,0)(0,1,0)	0.25	0.51	10.81	16.30	18.00	0.57	1
Ilam	ARIMA(0,0,0)(0,1,0)	0.79	0.54	8.81	21.75	18.00	0.24	1
Boshehr	ARIMA(0,0,0)(0,1,0)	0.36	0.72	7.95	21.08	18.00	0.28	1
Tehran	Winters' Additive	0.82	0.83	12.42	30.43	15.00	0.01	0
Chahrmahal_bakhtiari	ARIMA(0,0,0)(0,0,0)	0.99	0.99	8.60	20.39	18.00	0.31	4
Khorasan_Jonobi	Simple Seasonal	0.65	0.49	8.17	6.76	16.00	0.98	0
Khorasan_Razavi	Winters' Additive	0.72	0.67	11.58	11.41	15.00	0.72	0
Khorasan_Shonali	ARIMA(0,0,0)(0,0,0)	0.00	0.00	9.08	12.76	18.00	0.81	0
Khozestan	Winters' Additive	0.73	0.74	10.92	20.81	15.00	0.14	0
Zanjan	ARIMA(0,0,0)(0,0,0)	0.96	0.96	9.02	14.37	18.00	0.71	3
Semnan	ARIMA(0,1,1)(1,0,0)	0.76	0.52	7.89	18.21	16.00	0.31	1
Sistan_Balochestan	ARIMA(0,0,1)(0,1,0)	0.60	0.54	11.55	13.69	17.00	0.69	1
Fars	ARIMA(0,0,0)(0,1,0)	0.30	0.47	10.70	19.22	18.00	0.38	1
Ghazvin	ARIMA(0,0,0)(0,0,0)	0.00	0.00	9.44	13.92	18.00	0.73	0
Ghom	Winters' Additive	0.78	0.76	7.91	36.53	15.00	0.00	0

Kordestan	ARIMA(0,0,0)(0,1,0)	0.92	0.83	9.67	14.40	18.00	0.70	2
Kerman	ARIMA(0,0,0)(1,1,0)	0.61	0.72	10.57	16.33	17.00	0.50	1
Kermanshah	ARIMA(0,0,0)(0,0,0)	0.32	0.32	9.68	16.81	18.00	0.54	1
Kohgiloye_bouirahmad	ARIMA(2,0,0)(0,0,0)	0.98	0.98	7.79	16.82	17.00	0.47	2
Golestan	ARIMA(0,0,0)(0,1,0)	0.76	0.66	9.66	8.46	18.00	0.97	1
Gilan	ARIMA(0,0,0)(0,0,1)	0.50	0.50	10.88	5.72	17.00	1.00	1
Lorestan	ARIMA(0,0,0)(0,0,0)	0.85	0.85	9.35	20.97	18.00	0.28	1
Mazandaran	Winters' Additive	0.63	0.58	9.92	16.12	15.00	0.37	0
Markazi	ARIMA(0,0,0)(0,0,0)	0.73	0.73	9.15	16.32	18.00	0.57	2
Hormozgan	Simple Seasonal	0.72	0.68	9.09	25.90	16.00	0.06	0
Hamedan	ARIMA(0,0,0)(0,0,0)	0.68	0.68	10.19	16.91	18.00	0.53	2
Yazd	Simple Seasonal	0.74	0.44	8.31	14.67	16.00	0.55	0
**Male National (Iran)**	ARIMA(1,0,0)(0,1,0)	0.71	0.86	13.97	9.98	17.00	0.90	1
Male Azarbaijan_Sharghi	ARIMA(0,0,0)(0,0,0)	0.32	0.32	9.86	11.64	18.00	0.87	1
Male Azarbaijan_Gharbi	ARIMA(0,0,0)(0,0,0)	0.69	0.69	8.43	16.44	18.00	0.56	3
Male Ardebil	ARIMA(0,1,0)(0,0,0)	0.26	0.20	7.72	14.76	18.00	0.68	1
Male Esfahan	ARIMA(0,0,0)(1,0,0)	0.49	0.49	10.18	18.55	17.00	0.36	1
Male Alborz	ARIMA(0,1,0)(0,0,0)	0.32	0.45	9.54	30.30	18.00	0.04	1
Male Ilam	Simple Seasonal	0.72	0.31	6.27	11.75	16.00	0.76	0
Male Boshehr	Winters' Additive	0.61	0.64	7.54	11.21	15.00	0.74	0
Male Tehran	ARIMA(0,0,0)(0,1,0)	0.19	0.65	11.68	24.50	18.00	0.14	1
Male Chahrmahal_bakhtiari	ARIMA(0,0,0)(0,0,0)	0.86	0.86	7.83	32.06	18.00	0.02	3
Male Khorasan_Jonobi	Simple Seasonal	0.61	0.42	7.19	8.27	16.00	0.94	0
Male Khorasan_Razavi	ARIMA(0,0,0)(1,0,0)	0.43	0.43	10.67	12.97	17.00	0.74	1
Male Khorasan_Shonali	ARIMA(0,0,0)(0,0,0)	0.00	0.00	7.38	15.02	18.00	0.66	0
Male Khozestan	Winters' Additive	0.68	0.67	9.94	16.79	15.00	0.33	0
Male Zanjan	ARIMA(0,0,0)(0,0,0)	0.75	0.75	7.72	15.01	18.00	0.66	2
Male Semnan	Holt	0.83	0.23	6.98	13.54	16.00	0.63	0
Male Sistan_Balochestan	ARIMA(0,0,0)(0,1,0)	0.63	0.48	10.17	18.53	18.00	0.42	2
Male Fars	Simple Seasonal	0.54	0.51	9.40	16.48	16.00	0.42	0
Male Ghazvin	ARIMA(0,0,0)(0,0,0)	0.33	0.33	7.90	11.89	18.00	0.85	1

Male Ghom	ARIMA(0,1,0)(0,0,0)	0.32	0.06	7.42	21.26	18.00	0.27	1
Male Kordestan	ARIMA(0,0,0)(0,0,0)	0.00	0.00	8.32	11.12	18.00	0.89	0
Male Kerman	Winters' Additive	0.73	0.72	9.12	12.69	15.00	0.63	0
Male Kermanshah	ARIMA(0,0,0)(0,0,0)	0.65	0.65	8.31	19.70	18.00	0.35	2
Male Kohgiloye_bouirahmad	ARIMA(0,0,0)(0,0,0)	0.58	0.58	7.08	20.37	18.00	0.31	1
Male Golestan	Winters' Additive	0.77	0.54	8.32	19.59	15.00	0.19	0
Male Gilan	Simple Seasonal	0.77	0.34	9.00	29.99	16.00	0.02	0
Male Lorestan	ARIMA(1,0,0)(0,0,0)	0.53	0.53	7.96	14.55	17.00	0.63	1
Male Mazandaran	Winters' Additive	0.59	0.48	8.65	18.98	15.00	0.22	0
Male Markazi	ARIMA(0,0,0)(0,0,0)	0.37	0.37	7.49	18.75	18.00	0.41	1
Male Hormozgan	Simple Seasonal	0.69	0.62	8.22	19.86	16.00	0.23	0
Male Hamedan	Simple Seasonal	0.78	0.19	8.68	13.57	16.00	0.63	0
Male Yazd	ARIMA(0,1,0)(0,1,1)	0.95	0.88	6.74	15.04	17.00	0.59	4
**Female National (Iran)**	ARIMA(0,0,0)(0,1,0)	0.80	0.69	15.06	30.73	18.00	0.03	2
Female Azarbaijan_Sharghi	ARIMA(0,0,0)(0,0,0)	0.89	0.89	8.92	15.02	18.00	0.66	4
Female Azarbaijan_Gharbi	ARIMA(0,0,0)(0,0,0)	0.96	0.96	9.15	20.37	18.00	0.31	3
Female Ardebil	ARIMA(1,0,0)(0,0,0)	0.50	0.50	8.73	12.63	17.00	0.76	1
Female Esfahan	Simple Seasonal	0.61	0.43	9.88	15.74	16.00	0.47	0
Female Alborz	Winters' Additive	0.72	0.77	8.65	13.12	15.00	0.59	0
Female Ilam	ARIMA(0,0,0)(0,0,0)	0.94	0.94	6.84	21.65	18.00	0.25	2
Female Boshehr	ARIMA(1,0,0)(0,1,0)	0.76	0.84	6.53	11.89	17.00	0.81	2
Female Tehran	Winters' Additive	0.84	0.83	10.86	34.00	15.00	0.00	0
Female Chahrmahal_bakhtiari	ARIMA(0,0,0)(0,0,0)	1.00	1.00	6.94	15.55	18.00	0.62	5
Female Khorasan_Jonobi	Simple Seasonal	0.80	0.42	6.92	12.19	16.00	0.73	0
Female Khorasan_Razavi	ARIMA(0,0,0)(1,0,0)	0.40	0.40	11.01	14.79	17.00	0.61	1
Female Khorasan_Shonali	Simple Seasonal	0.52	0.16	8.34	11.07	16.00	0.81	0
Female Khozestan	Winters' Additive	0.78	0.78	9.25	25.10	15.00	0.05	0
Female Zanjan	ARIMA(2,0,0)(0,0,0)	0.98	0.98	8.16	6.30	17.00	0.99	3
Female Semnan	ARIMA(0,0,0)(0,0,0)	0.51	0.51	6.71	27.43	18.00	0.07	1

Female Sistan_Balochestan	ARIMA(0,1,0)(0,1,0)	0.49	0.70	9.94	22.75	18.00	0.20	2
Female Fars	ARIMA(0,0,0)(0,1,0)	0.62	0.65	9.34	14.52	18.00	0.70	2
Female Ghazvin	ARIMA(0,0,0)(0,0,0)	0.40	0.40	8.36	13.18	18.00	0.78	1
Female Ghom	ARIMA(0,0,0)(1,1,0)	0.60	0.80	6.75	25.98	17.00	0.08	1
Female Kordestan	ARIMA(0,0,0)(0,0,0)	0.97	0.97	8.03	19.91	18.00	0.34	4
Female Kerman	ARIMA(0,0,0)(0,1,0)	0.74	0.64	9.60	22.68	18.00	0.20	1
Female Kermanshah	ARIMA(0,0,0)(0,0,0)	0.00	0.00	8.54	18.66	18.00	0.41	0
Female Kohgiloye_bouirahmad	ARIMA(0,0,0)(0,1,0)	0.99	0.98	7.07	22.81	18.00	0.20	2
Female Golestan	ARIMA(0,0,0)(0,0,0)	0.66	0.66	8.91	23.89	18.00	0.16	2
Female Gilan	ARIMA(0,0,0)(0,0,0)	0.55	0.55	10.05	9.04	18.00	0.96	1
Female Lorestan	ARIMA(0,0,0)(0,0,0)	0.91	0.91	8.34	11.19	18.00	0.89	1
Female Mazandaran	ARIMA(0,0,0)(0,1,0)	0.41	0.44	8.94	34.55	18.00	0.01	1
Female Markazi	ARIMA(0,0,0)(0,1,0)	0.92	0.82	8.22	23.52	18.00	0.17	1
Female Hormozgan	Simple Seasonal	0.71	0.66	7.54	26.60	16.00	0.05	0
Female Hamedan	ARIMA(0,0,0)(0,0,0)	0.89	0.89	9.05	12.15	18.00	0.84	3
Female Yazd	ARIMA(1,0,0)(0,0,0)	0.49	0.49	7.24	11.16	17.00	0.85	1

Footnote: SD: standard deviation, DF: degree of freedom, BIC: Bayesian information criterion. (p, d, q) is the non-seasonal part of the ARIMA model which shows auto-regressive order, the degree of differencing, and the moving average order, respectively. (sp, sd, sq) is the seasonal part of the ARIMA model which indicates seasonal auto-regressive order, seasonal degree of differencing, seasonal moving average order, respectively.
