# Supplementary material for: Excess all-cause mortality and COVID-19 reported fatality in Iran (April 2013–September 2021): age and sex disaggregated time series analysis
Source: BMC Res Notes. 2022 Apr 5;15:130. doi: 10.1186/s13104-022-06018-y (PMC8981187; doi:10.1186/s13104-022-06018-y)
Supplement: Supplementary file 2 — Additional file 2: Table S2. The seasonal excess death rate, male to female ratio, and COVID-19 reported deaths coverage from winter 2020 to summer 2021. Table S3. Excess deaths at the province level in a year from summer 2020 until spring 2021 in Iran. [file 13104_2022_6018_MOESM2_ESM.pdf]

Table S2. Seasonal excess death among male and females and COVID-19 reported deaths coverage.

	Observed/Expected (percent change)	COVID-19 reported death (% of all cause excess death)	Male excess death/ Female excess death (M:F ratio)	Excess deaths per 100,000 population (95% CI)
Winter 2020	106480/100881 (5.6%)	1284 (22.9%)	3787/1450 (2.6)	6.69 (-1.94-15.31)
Spring 2020	108623/90492 (20%)	8223 (45.4%)	10127/7530 (1.3)	21.65 (13.02-30.28)
Summer 2020	131235/91273 (43.8%)	14971 (37.5%)	21859/17697 (1.2)	47.72 (39.09-56.34)
Autumn 2020	156857/103133 (52.1%)	29147 (54.3%)	34951/23507 (1.5)	64.15 (55.52-72.78)
Winter 2021	111909/100881 (10.9%)	8099 (73.4%)	5542/4702 (1.2)	13.17 (0.97-25.37)
Spring 2021	135569/90491 (49.8%)	21377 (47.4%)	23158/21007 (1.1)	53.83 (41.62-66.03)
Summer 2021	162570/91273 (78.1%)	30519 (42.8%)	37396/33068 (1.1)	85.13 (97.33-72.93)
summer 2020 – spring 2021 Total	535570/385778 (38.8%)	73594 (49.1%)	85510/66913 (1.3)	178.86 (137.20-220.51)

Table S3. Excess deaths at the province level from summer 2020 until spring 2021 in Iran.

Province	Observed/Expected death count (percent change)	ED rate (95% CI)	Male ED rate (95% CI)	Female ED rate (95% CI)
Azerbaijan Sharghi	33439/22352 (49.6%)	273.2 (224.5-322)	307.8 (259.2-356.6)	263.1 (236.5-289.8)
Hamedan	14846/10304 (44.1%)	263.7 (198.7-329)	263.8 (199-328.4)	252.8 (189.7-316)
Zanjan	7848/5160 (52.1%)	246.4 (192.1-300.3)	252.4 (193.3-310.7)	229.7 (156.5-302.7)
Markazi	11161/7820 (42.7%)	231.7 (185.6-278)	238.5 (196.5-280.5)	233.2 (157.8-308.6)
Ghazvin	8877/5864 (51.4%)	226.3 (161.1-291.5)	245 (189.4-300)	224.5 (155.4-293.5)
Kordestan	11231/7422 (51.3%)	225.3 (160.2-290.4)	259.5 (202.5-317.1)	199.5 (158.9-240.5)
Azerbaijan Gharbi	22831/15394 (48.3%)	217.9 (168.7-267.1)	245.5 (220.2-270.7)	236 (198.2-273.5)
Ardebil	9862/7135 (38.2%)	211.7 (110.1-313.6)	167.5 (51.2-283.7)	234 (136.1-331.9)
Lorestan	12225/8616 (41.9%)	204.4 (160-248.5)	215.4 (167.7-263.2)	188.8 (134.7-243.3)

Kermanshah	14968/11065 (35.3%)	199.3 (152.1-246.5)	211.5 (170-253)	178.7 (121.1-235.9)
Alborz	17691/11820 (49.7%)	198.8 (132.8-264.8)	241.2 (112.9-369.5)	178.7 (142.3-215.1)
Tehran	86140/60496 (42.4%)	181.4 (156.9-206)	233.6 (188.4-278.8)	153.9 (131.2-176.5)
Fars	31544/22477 (40.3%)	179.4 (142.8-215.9)	201 (162.1-239.8)	169.1 (133.7-204.4)
**Iran**	535570/385778 (38.8%)	178.9 (137.2-220.5)	200.9 (175.1-226.7)	162.5 (122.9-202.2)
Khuzestan	29596/21208 (39.6%)	172.8 (139-206.6)	192 (151.3-232.7)	152.8 (122.9-182.8)
Esfahan	35088/25935 (35.3%)	172.2 (131.1-213.3)	248.1 (204.3-291.9)	169 (124.3-213.6)
Chaharmahal Bakhtiari	5811/4120 (41%)	170.8 (130.2-211.4)	175.4 (114.7-236.1)	169.3 (135.1-203.5)
Khorasan Shomali	5989/4532 (32.1%)	169.5 (85.2-253.7)	179.7 (108.9-250.4)	79.1 (-103.4-261.6)
Ilam	3538/2527 (40%)	169 (49.3-289)	209.7 (153.4-265.1)	142.2 (78-205.8)
Semnan	4848/3664 (32.3%)	155.9 (97.3-214.6)	190.5 (128.4-252.4)	175.7 (118.9-231.5)
Yazd	6916/5089 (35.9%)	153.5 (100.1-206.9)	52.1 (-24.3-128.1)	154.8 (98.4-211.4)
Khorasan Razavi	42296/32184 (31.4%)	149 (115.4-182.6)	204.7 (162.6-246.8)	149.7 (99.1-200.4)
Golestan	11789/9040 (30.4%)	141.5 (85.1-198)	130 (84.4-175.6)	166.7 (105.2-228.2)
Kohgiloye Bouirahmad	3813/2756 (38.4%)	139.7 (90.4-189)	158 (93-222.1)	111.3 (30.1-192.5)
Ghom	8157/6243 (30.7%)	136.2 (110.3-162)	144.4 (51.4-237.3)	122.2 (95.5-148.7)
Bushehr	6011/4414 (36.2%)	125.9 (86.6-165.2)	119.9 (74.6-165.1)	128.2 (86.9-169.2)
Gilan	19504/16292 (19.7%)	125 (60.4-189.5)	128.5 (77.2-179.8)	129.9 (42.9-217.1)
Khorasan Jonobi	4950/3903 (26.8%)	122.5 (70.9-174.2)	142.2 (79.8-204.2)	102.3 (46.1-158.7)
Kerman	18473/14445 (27.9%)	120.4 (80.2-160.5)	128.7 (90.2-167.3)	97.1 (35.5-158.9)
Sistan Balochestan	15966/12540 (27.3%)	115.4 (16.9-214.1)	131.8 (49.2-214.2)	124.5 (-54.8-303.8)
Mazandaran	21713/17953 (20.9%)	108.9 (75.1-142.7)	108.1 (72.8-143.3)	137.2 (89.3-185.1)
Hormozgan	8458/6539 (29.3%)	99.2 (63.1-135.2)	109.7 (64-155.3)	88.2 (54-122.3)
