# Supplementary material for: Excess all-cause mortality and COVID-19 reported fatality in Iran (April 2013–September 2021): age and sex disaggregated time series analysis
Source: BMC Res Notes. 2022 Apr 5;15:130. doi: 10.1186/s13104-022-06018-y (PMC8981187; doi:10.1186/s13104-022-06018-y)

Figure S2. Weekly all-cause death, COVID-19 reported death, and COVID-19 reported cases from 23 March 2019 to 18 September 2021.

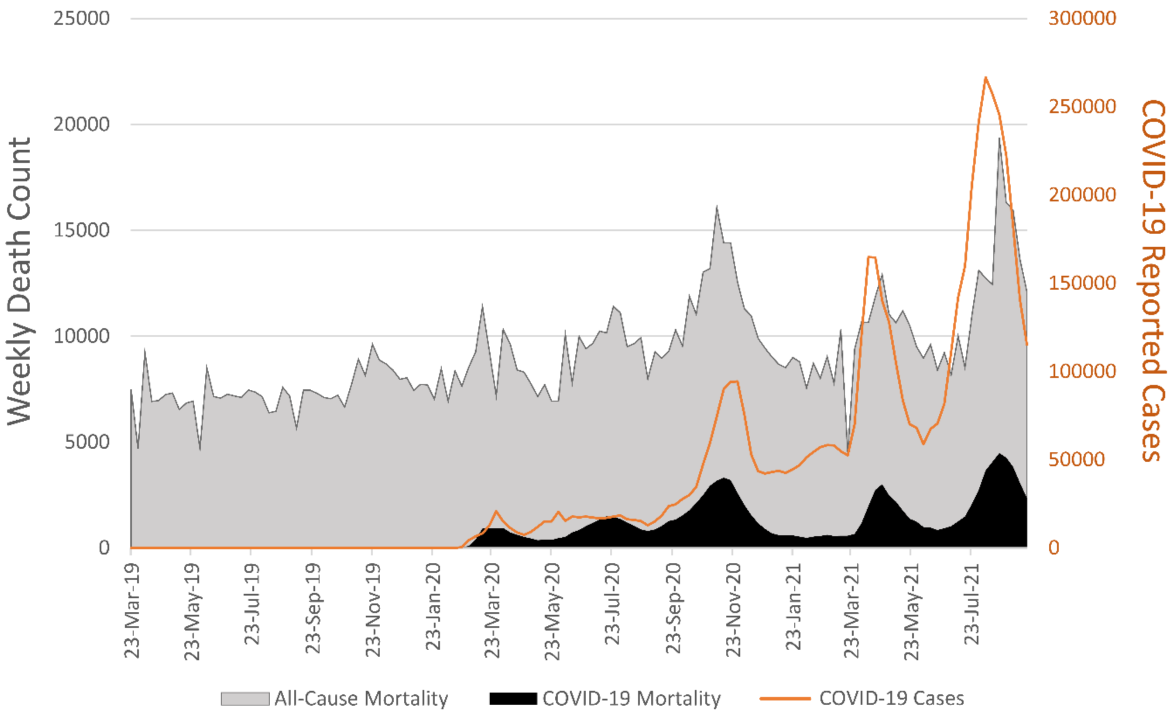

Figure S3. Relation of weekly all-cause death and COVID-19 reported mortality during the COVID-19 pandemic in Iran (From March 2020 to August 2021).

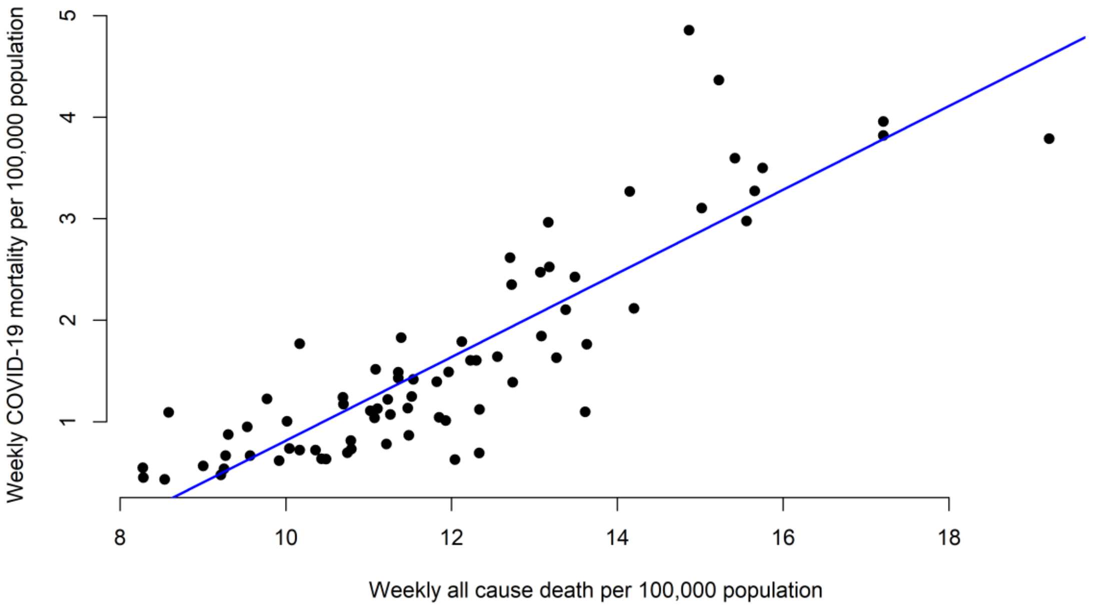

Supplement: Supplementary file 3 — Additional file 3: Figure S2. Weekly all-cause death, COVID-19 reported death, and COVID-19 reported cases from 23 March 2019 to 22 September 2021. Figure S3. Relation of weekly all-cause death and COVID-19 reported mortality during the COVID-19 pandemic in Iran (From March 2020 until September 2021) [file 13104_2022_6018_MOESM3_ESM.pdf]
